# Effectiveness of the Internet of Things for Improving Working-Aged Women’s Health in High-Income Countries: Protocol for a Systematic Review and Network Meta-analysis

**DOI:** 10.2196/45178

**Published:** 2023-04-04

**Authors:** Noyuri Yamaji, Aya Nitamizu, Etsuko Nishimura, Daichi Suzuki, Kiriko Sasayama, Md. Obaidur Rahman, Eiko Saito, Daisuke Yoneoka, Erika Ota

**Affiliations:** 1 Global Health Nursing, Graduate School of Nursing St. Luke’s International University Tokyo Japan; 2 Department of Nursing, Faculty of Health and Medical Sciences Kanagawa Institute of Technology Kanagawa Japan; 3 Global Health Nursing International University of Health and Welfare Chiba Japan; 4 Center for Surveillance, Immunization, and Epidemiologic Research National Institute of Infectious Diseases Tokyo Japan; 5 Center for Evidence-Based Medicine and Clinical Research Dhaka Bangladesh; 6 Institute for Global Health Policy Research National Center for Global Health and Medicine Tokyo Japan; 7 Tokyo Foundation for Policy Research Tokyo Japan

**Keywords:** Internet of Things, IoT, women’s health, network meta-analysis, apps, meta analysis, meta analyses, review method, systematic review, high income, mHealth, mobile health, health app, application, digital health

## Abstract

**Background:**

Women often experience many unique health issues and conditions throughout their working lives. The Internet of Things (IoT) is a system of interrelated digital devices that can enable data exchanges over a network without human-to-human or human-to-computer interaction. The usage of applications and IoT in improving women’s health has recently increased worldwide. However, there has been no consensus on the effectiveness of IoT in improving women’s health outcomes.

**Objective:**

This systematic review and network meta-analysis (NMA) aims to assess and synthesize the role of apps and the IoT in improving women’s health and to identify the ranking of interventions for ensuring better results for each stated outcome.

**Methods:**

Our systematic review and NMA will be conducted in accordance with the guidelines of the Cochrane Handbook. We will comprehensively search the following electronic databases: PubMed (including MEDLINE), Cochrane Central Register of Controlled Trials, Embase, Cumulative Index to Nursing and Allied Health Literature (ie, CINAHL), PsycINFO, ClinicalTrials.gov, and the World Health Organization International Clinical Trials Registry, along with other resources to identify relevant randomized controlled trials that have assessed the effects of various apps and the IoT with regard to improving working-aged women’s health in high-income countries. We will segment and analyze the results of the included studies based on age categories (women undergoing a preconception period, those undergoing gestational and postpartum periods, and menopausal and pre- and postmenopausal women) and the medical history (women who have a specific medical condition—eg, cancer or diabetes—and women who do not have them) separately. Two independent reviewers will perform the study selection, data extraction, and quality assessment. Our primary outcomes include health status, well-being, and quality of life. We will perform pairwise meta-analysis and NMA to estimate the direct, indirect, and relative effects of apps and the IoT on women’s health outcomes. We will also assess the hierarchy of interventions, statistical inconsistencies, and certainties of evidence for each outcome.

**Results:**

We plan to conduct the search in January 2023 and are currently discussing search strategies with the literature search specialists. The final report is planned for submission to a peer-reviewed journal in September 2023.

**Conclusions:**

To the best of our knowledge, this review will be the first to identify the ranking of IoT intervention for ensuring working-aged women’s health outcomes. These findings may be of great use to researchers, policy makers, and others with an interest in the field.

**Trial Registration:**

International Prospective Register of Systematic Reviews (PROSPERO) CRD42022384620; https://www.crd.york.ac.uk/prospero/display_record.php?RecordID=384620

**International Registered Report Identifier (IRRID):**

PRR1-10.2196/45178

## Introduction

Since the 1990s, the global labor force participation rate has been decreasing gradually. The Asia-Pacific region has experienced an overall decline in labor participation, which is believed to be mainly responsible for the global decline [1]. However, for regions outside of Africa, it has been estimated that the labor force participation rate will decrease, and this trend is projected to continue until at least 2030 [1,2]. Although women are less likely to participate in the labor market than men [1], women’s participation has increased gradually owing to increased education and employment opportunities [3].

However, certain lifestyle changes, which occurred because of women’s social advancement, have affected their health. For instance, women often perceive their health to be worse than do men because of certain social and biological factors [4], and consistently report higher stress levels [5]. A delay in marriage and the increasing age of conception have been correlated with higher chances of infertility in women, and over 186 million people (both men and women) have experienced infertility worldwide [6]. As opposed to primary infertility, secondary infertility is mainly related to lifestyle-related risks. A previous review has provided evidence of an association between lifestyle behaviors and infertility, including obesity, stress, a disturbed circadian clock, smoking, alcohol, and exercise [7]. Women’s social advancement is associated with changes in their daily lives. Compared to working men, the double burden of job demands and domestic responsibilities has made women vulnerable to developing noncommunicable diseases [8].

Moreover, women experience considerable hormonal changes, unique health issues, and conditions throughout their lives [9,10]. Life stages of working-aged women are generally divided into reproductive age, pregnancy and delivery, and a climacteric period [9]. They may experience many unique health issues and conditions throughout their lives (eg, from preconception periods and menopause to several gynecological conditions) [11,12]. The prevalence of obesity in women of reproductive age has been increasing worldwide. Furthermore, it is associated with the risk of reduced fertility and the development of obesity-related comorbidities [13]. During pregnancy, maternal medical conditions, including hypertension, diabetes, and obesity, increase the risk of adverse outcomes, such as preterm delivery, low birth weight, and infant death [14-16]. Furthermore, the development of maternal medical conditions is associated with a risk of developing chronic disease later in life [13,17]. For example, women who develop gestational diabetes have a higher risk of developing type 2 diabetes in the future [17]. Women in the climacteric periods are exposed to the risk of developing chronic diseases, such as obesity, metabolic syndrome, diabetes, cardiovascular disease, depression, and cancer [18]. These diseases are associated with their lifestyle habits [9,18,19]. Lifestyle factors can impact the gynecological well-being of women throughout their lives and will affect the future health of subsequent generations [20]. Thus, it is necessary to strive to prevent and improve common medical conditions in women, taking into account their lifestyles throughout the overall life stages.

While women live longer than men [21], women are more affected and bear a heavier burden of living with disability [22]. Ensuring women’s health and control over their own fertility can contribute to economic development; furthermore, ensuring women’s health is important for ensuring the health and economic well-being of subsequent generations [23]. In summary, continuous care throughout women’s lives is necessary, which, in turn, is important for maintaining community- and national-level economic development.

In 2022, a total of 5 billion people (63% of the global population) used the internet, and this figure continues to increase annually [24]. In 2009, the concept of smart health care was introduced. Since then, attempts to use the Internet of things (IoT) and other technologies to manage information related to people's health and address health care have been expanded [25]. IoT is a system of interrelated digital devices that can enable data exchanges over a network without human-to-human or human-to-computer interactions [26,27]. The IoT is increasingly being used to ensure and improve women’s health worldwide (eg, monitoring [28-30] and providing interventions [30,31]). The IoT, which can be used daily and continually without human interactions, could successfully provide the opportunities and motivation necessary for women to engage in good lifestyle behaviors. Among IoT interventions, smart health care systems are used for health prevention and improvement. Smart health care is mainly used in home care, self-care, and acute care settings, where self-care systems allow people to monitor their own health conditions and access that information through wearable devices and smartphones [26,32]. Working-aged women would use these devices throughout the day, including at work and at home, to manage their lifestyles on their own. Previous studies have revealed the effectiveness of IoT in encouraging lifestyle behavioral changes and improving people's health. A previous systematic review has shown that personalized mobile interventions for people (64% of which were women) improved lifestyle behaviors [33]. Some primary studies have reported that interventions that use IoT have improved women’s lifestyles [34-36] and health outcomes (eg, adverse pregnancy outcomes [35] and markers indicating hemodynamic functions [36]). However, there are various types of IoT interventions, such as exercise, diet, sleep, and combinations of these. Nonetheless, there are no systematic reviews assessing the effectiveness of the IoT among working-aged women, and no true consensus about the effectiveness of the IoT in improving women’s health outcomes has been established [33,37]. Systematic reviews and network meta-analyses (NMAs) can compare 3 or more interventions simultaneously on the basis of the results of existing primary studies in a single analysis and provide an overall statistic and up-to-date summary of the state of research knowledge on an intervention [38]. Therefore, a rigorous evaluation that considers all forms of the IoT is necessary for generating evidence and promoting appropriate integration and usage of technologies within existing health systems in order to improve women’s health outcomes.

## Methods

### Study Design

This systematic review and NMA aims to assess and synthesize the role of IoT in improving women’s health and to identify the ranking of interventions of apps and IoT for ensuring better results among women in high-income countries. NMA can combine any direct and indirect evidence into a single effect size [38]. Our NMAs will estimate the direct, indirect, and relative effects of IoT on women’s health outcomes and identify the ranking of interventions for ensuring the best results for each stated outcome.

This protocol adheres to the guidelines of the Cochrane Handbook for Systematic Reviews of Interventions [38] and reports its review findings based on the Preferred Reporting Items for Network Meta-Analyses (PRISMA-NMA) extension statement for reporting systematic reviews incorporating NMA of health care interventions [39]. We will report the results of each population group (women who are undergoing their preconception period, women undergoing their gestational and postpartum periods, and menopausal and pre- and postmenopausal women) separately. The review protocol is registered under the “International Prospective Register for Systematic Reviews” (PROSPERO) database (CRD42022384620) [40].

### Inclusion and Exclusion Criteria

This study used the PICOS framework (P: participants, I: interventions, C: comparison, O: outcomes, and S: study design) to determine the eligibility criteria for including studies in its review.

#### Participants

The study participants are working-aged women in high-income countries, as defined by the World Bank in 2023 [41]. To prevent heterogeneity in the objectives and settings of IoT interventions, we focused on high-income countries because the global burden of disease differs across high-income and low- and middle-income countries [42]. The Organization for Economic Co-operation and Development (ie, OECD) has defined the working-age population as those aged from 15 to 64 years [43]. We will segment and analyze studies based on women who are undergoing their preconception period, women undergoing their gestational and postpartum periods, and menopausal and pre- and postmenopausal women. This study will include women of reproductive age (15-49 years old) as women in the preconception period. It will also include middle-aged women (40-64 years) as menopausal and pre- and postmenopausal women. Although this study’s inclusion criteria show some overlap between the age categories, we will segment on the basis of the purposes of IoT interventions and populations of the included studies. Regarding studies focused on mixed populations (eg, those that include men), we will include only those studies that separate the data based on gender or those that include over 80% of women participants who meet our inclusion criteria.

#### Intervention and Comparison

We will include studies that have investigated IoT interventions to improve women’s health compared to the use of standard care, no intervention, or another intervention not using the IoT (eg, education or exercise without the IoT). The IoT in the medical field includes functions such as relaying medical information through mobile health or telemedicine, collecting data, and monitoring. Although there are various types of IoT, we will include the application areas, such as collecting and monitoring data with the sensors of smartphones and wearable devices, as the form of IoT. We will include studies that have targeted both male and female populations if the relevant IoT intervention has been designed to improve women’s health. If studies have investigated combinations of IoT interventions with other modes, we will also include studies that revealed IoT intervention as the primary intervention component.

#### Outcomes

##### Primary Outcomes

The outcomes for all included populations will be health status, including the number of cases diagnosed or treated (eg, cases of obesity and mental disorder), well-being, and quality of life.

Outcomes for women in their preconception periods include live birth (defined as the delivery of a live fetus after 20 completed weeks of gestation) or ongoing pregnancy (defined as evidence of a gestational sac with fetal heart motion at 12 weeks, as confirmed through ultrasonography.

Outcomes for women in the gestational and postpartum periods include health status, including the number of cases diagnosed or treated, including those of high-risk pregnancy (eg, gestational hypertension, gestational diabetes, and preterm birth).

Neonatal outcomes include low birth weight (defined as a birth weight less than 2.5 kg) and perinatal mortality.

Outcomes for middle-aged women include health status, including the number of cases diagnosed or treated (eg, diabetes, metabolic syndrome, hyperlipidemia, and hypertension).

##### Secondary Outcomes

Outcomes for all included populations are lifestyle and behavioral changes, including maintaining a healthy weight (measured as BMI in kg/m², % weight loss, or number of people with a BMI between 18.5 and 25 kg/m² [2]), maintaining healthy sleep behavior (sleeping 7 or more hours per night on a regular basis), stopping or reducing alcohol intake (measured as alcoholic drinks per day, with 1 drink of >10 g of ethanol), increasing physical activity (in accordance with the World Health Organization’s standards; measured as minutes of moderate to vigorous physical activity per week or number of people reaching the World Health Organization’s recommendation of doing 150 minutes of moderate‐intensity physical activity per week, preferably based on the Global Physical Activity Questionnaire or other validated scales), and quitting or ceasing smoking and other substances (measured as the number of people not smoking or the number of people not using other substances).

If the studies report outcomes across different time points, this review will select the time point at the end of the relevant intervention.

#### Types of Study

We will include individual randomized controlled trials (RCTs) and cluster-RCTs to investigate the effects of IoT interventions on women’s health. We will exclude reviews, qualitative studies, observational studies, cross-sectional studies, case studies, commentaries, editorials, expert opinions, and letters.

### Searching the Literature

We will perform a comprehensive search to identify any relevant studies that meet our eligibility criteria by using the following electronic databases: PubMed (including MEDLINE), Cochrane Central Register of Controlled Trials, Embase, Cumulative Index to Nursing and Allied Health Literature (ie, CINAHL), and PsycINFO; there will be no restrictions on languages and publication dates. We will search ClinicalTrials.gov (clinicaltrials.gov) and the World Health Organization International Clinical Trials Registry (apps.who.int/trialsearch/) to identify additional ongoing studies. We will develop search strategies for each electronic database addressing the review question. The search strategies will use keywords and controlled vocabulary as follows: participants: *woman*, interventions: *IoT* (eg, *mobile application*, *wearable electronic device*, and *smart device*), and study design: *RCT*. We will also check the reference lists of included reports and relevant systematic reviews to identify additional potential studies. The complete review will report the detailed search strategies for all electronic databases.

### Study Selection

**Figure 1:**
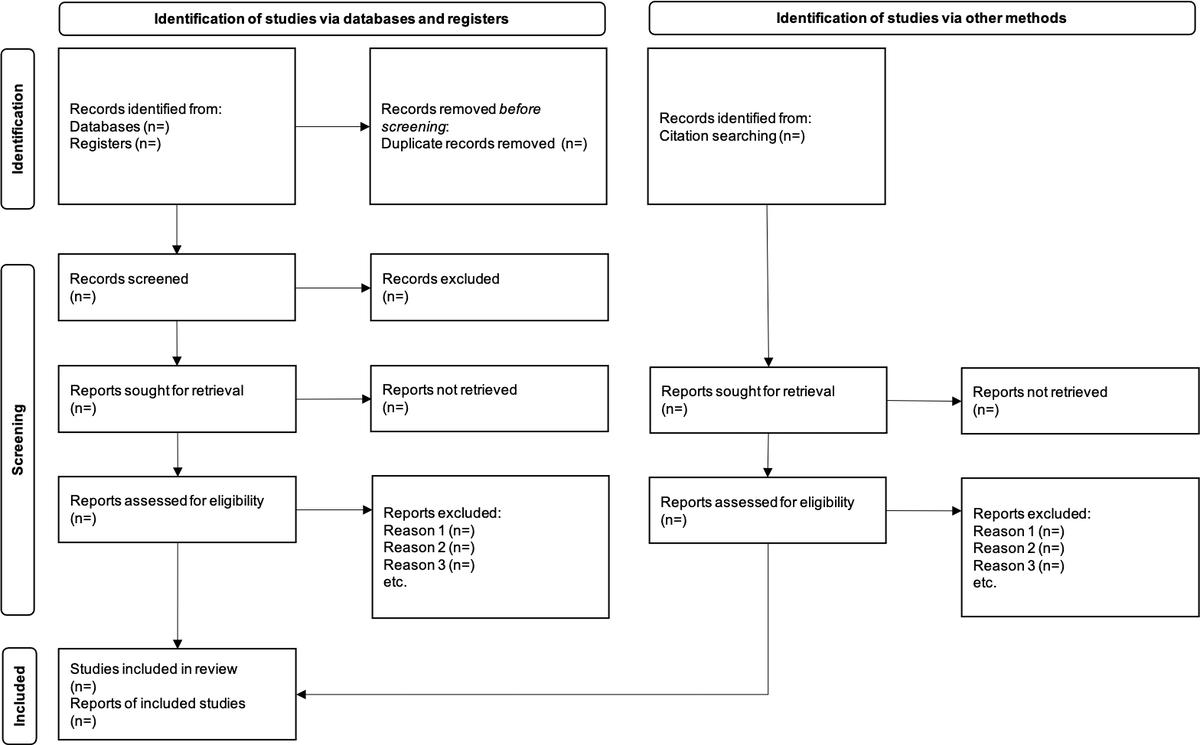
Preferred Reporting Items for Systematic Reviews and Meta-Analyses flow diagram.

We will screen the relevant studies in accordance with the PRISMA guidelines (the PRISMA flow diagram is shown in Figure 1) [44]. First, we will exclude any duplicate studies. Two reviewers will independently screen the titles and abstracts of all the retrieved studies. Furthermore, 2 authors will independently assess the full texts of all potentially eligible studies. The Rayyan tool will be used for screening during the study selection. Any disagreements between reviewers will be resolved through discussion. If an agreement is not reached, we will discuss the issue with a third reviewer to make the final decision. We will also manage the selected studies by using the bibliographic citation management software EndNote X9.

### Data Extraction

Two authors will independently extract data from each of the included studies using a predesigned data extraction form using Excel (Microsoft Inc). We will extract data regarding the characteristics of included studies (eg, study setting, participants, interventions, comparisons, study design, and outcome measures, including primary and secondary outcomes). Any discrepancies will be resolved through discussion with a third author. In case of any unclear or missing information, we will contact the authors to collect the relevant data.

### Risk of Bias Assessment

Two reviewers will independently assess the risk of bias for each outcome using the Cochrane Collaboration's risk of bias assessment tool 2.0 [45]. We will assess the following domains: those related to the randomization process, deviations from intended interventions, missing outcome data, measurement of the outcomes, and selection of the reported results. The overall risk of bias will be discerned as follows: low risk of bias, some concerns, or high risk of bias [45]. Any disagreement will be resolved through discussion with a third reviewer.

### Data Synthesis

We will perform the NMA on age categories (preconception periods, gestational and postpartum periods, and middle-aged women) and medical history (women who have a specific medical condition; eg, cancer or diabetes, and women who do not have them) separately. We will present the network structure of IoT interventions and the estimated direct, indirect, and relative effects for each outcome. First, we will conduct a pairwise meta-analysis using a fixed effects model to estimate the pooled effect size of all direct evidence for each combination of outcomes. In case of considerable heterogeneity, we will use a random effects (DerSimonian and Laird) model. Then, we will perform NMAs for primary and secondary outcomes to compare the individual IoT interventions. For dichotomous variables, we will use risk ratios or odds ratios and the corresponding 95% CIs. For continuous variables, we will calculate the mean difference or standardized mean difference using 95% CIs. If the measurements of intervention effects with regard to each outcome have been reported using different scales, we will use a standardized mean difference and 95% CIs. We will narratively describe the results if studies are insufficient for conducting a meta-analysis. Heterogeneity will be examined using the estimated value of τ and the chi-square test and assessed graphically using horizontal lines in the forest plots for each outcome. Furthermore, heterogeneity will be examined using *I*^2^ statistics, with a value greater than 75% indicating considerable heterogeneity [38]. For pairwise meta-analysis with an adequate number of included studies, publication bias will be tested using funnel plots and the Egger test; for NMA, we will use a comparison-adjusted funnel plot to identify possible-small study effects [46,47]. A surface under the cumulative ranking curve (SUCRA) will be used for ranking probabilities of interventions to identify the most effective intervention. A larger SUCRA value indicates a better rank of intervention [46]. A subgroup analysis for the primary outcome will be performed for the types of IoT (single approach and combination) and types of IoT devices (wearable devices worn on the body and other devices not worn). To assess the robustness of the results, sensitivity analysis will be performed, excluding studies at a high risk of bias.

### Inconsistency Assessment

We will assess any inconsistencies between direct and indirect evidence by using local and global approaches. Regarding the evaluation of global inconsistencies, we will demonstrate the design by using a treatment interaction model and infer the presence of any inconsistency based on a chi-square test. Regarding the evaluation of local inconsistencies, we will use the side-splitting method and assess the disagreement between direct and indirect effects. We will consider a *P* value of <.05 to indicate statistical significance.

### Certainty of Evidence Assessment

We will assess the certainty of the evidence for primary outcomes by using the Grades of Recommendation, Assessment, Development and Evaluation method for NMA [48]. We will assess the following factors: risk of bias, inconsistency, indirectness, imprecision, and publication bias; furthermore, we will classify the certainty of evidence as follows: high, moderate, low, or very low. We will also aim to assess the certainty of evidence for each of the direct estimates, indirect estimates, and relative estimates. If only direct or indirect evidence is available for a given comparison, we will consider their ratings as the network ratings. If both types of evidence are available, we will consider the higher ratings of the evidence as the network ratings. We will summarize the results for primary outcomes in a “summary of findings” table (Multimedia Appendix 1) [49].

## Results

We aim to conduct our search in January 2023 and are currently discussing search strategies with literature search specialists. We will estimate their relative ranking for women's health outcomes by conducting the NMA. This study is expected to identify the most effective IoT modality in improving women’s health outcomes. The final report is planned for submission to a peer-reviewed journal in September 2023.

## Discussion

To the best of our knowledge, this will be the first comprehensive systematic review and NMA of RCTs, which aims to assess and synthesize the role of apps and the IoT in improving women’s health and to identify the ranking of interventions for ensuring better results among women for each stated outcome. Recently, the number of women with issues and conditions related to their lifestyle habits has been increasing [8]. Problems with lifestyle habits are known to lead to the development of future noncommunicable diseases [7], and improving women's lifestyles will help prevent and improve their common medical conditions. As such, our systematic review’s findings will have the potential to influence a large proportion of the population, including women and their subsequent generations. These findings may be of great use to health care providers, policy makers, and others who have an interest in this field. Furthermore, our results can be extensively used in improving the health of women both presently and in subsequent generations.

## Appendix

Multimedia Appendix 1 Dummy table of summary of findings.

## Abbreviations

IoT: Internet of things
NMA: network meta-analysis
PRISMA-NMA: Preferred Reporting Items for Network Meta-Analyses
PROSPERO: International Prospective Register for Systematic Reviews
RCT: randomized controlled trial
SUCRA: surface under the cumulative ranking curve

## Data Availability

All data generated or analyzed during this study will be included in the full review article and its supplementary information files.
