# Supplementary material for: Effectiveness of the Internet of Things for Improving Working-Aged Women’s Health in High-Income Countries: Protocol for a Systematic Review and Network Meta-analysis
Source: JMIR Res Protoc. 2023 Apr 4;12:e45178. doi: 10.2196/45178 (PMC10131648; doi:10.2196/45178)
Supplement: Multimedia Appendix 1 [file resprot_v12i1e45178_app1.docx]

**Table S1.** Dummy table of summary of findings.

| **IoT compared to usual care, no intervention, and other interventions for women's health** | | | | | | |
| --- | --- | --- | --- | --- | --- | --- |
| **Population**: working-aged women  **Setting**: high-income countries  **Intervention**: internet of things (IoT)  **Comparison**: usual care, no intervention, and other interventions | | | | | | |
| **Outcomes** | **Anticipated absolute effects^*^ (95% CI)** | | **Relative effect (95% CI)** | **№ of participants (studies)** | **Certainty of the evidence (GRADE)** | **Comments** |
|  | **Risk with usual care, no intervention, and other interventions** | **Risk with IoT** |  |  |  |  |
| **Outcomes for working-aged women** | | | | | | |
| Obesity |  |  |  | (studies) | - |  |
| Mental disorder |  |  |  | (studies) | - |  |
| **Outcomes for women in their preconception periods** | | | | | | |
| Live birth |  |  |  | (studies) | - |  |
| Ongoing pregnancy |  |  |  | (studies) | - |  |
| **Outcomes for women in the gestational and postpartum periods** | | | | | | |
| Gestational hypertension |  |  |  | (studies) | - |  |
| Gestational diabetes |  |  |  | (studies) | - |  |
| Preterm birth |  |  |  | (studies) | - |  |
| Low birth weight |  |  |  | (studies) |  |  |
| **Outcomes for middle-aged women** | | | | | | |
| Diabetes |  |  |  | (studies) |  |  |
| Metabolic syndrome |  |  |  | (studies) |  |  |
| Hyperlipidemia |  |  |  | (studies) |  |  |
| Hypertension |  |  |  | (studies) |  |  |
| These outcomes were selected as the main outcomes of this study.  *The risk in the intervention group (and its 95% confidence interval) is based on the assumed risk in the comparison group and the relative effect of the intervention (and its 95% CI). CI: confidence interval | | | | | | |
| **GRADE Working Group grades of evidence** High certainty: we are very confident that the true effect lies close to that of the estimate of the effect. Moderate certainty: we are moderately confident in the effect estimate: the true effect is likely to be close to the estimate of the effect, but there is a possibility that it is substantially different. Low certainty: our confidence in the effect estimate is limited: the true effect may be substantially different from the estimate of the effect. Very low certainty: we have very little confidence in the effect estimate: the true effect is likely to be substantially different from the estimate of effect. | | | | | | |
